# Unveiling Gram-Ghost Cells: A Crucial Aspect of Gram Staining in the COVID-19 Era

**DOI:** 10.7759/cureus.54118

**Published:** 2024-02-13

**Authors:** Yoshiro Hadano

**Affiliations:** 1 Division of Infection Control and Prevention, Shimane University Hospital, Izumo, JPN

**Keywords:** gram-ghost cells, covid 19, covid, gram staining, mycobacterium tuberculosis complex

## Abstract

The term "Gram-ghost appearance" refers to mycobacteria's unique Gram staining characteristics. Recognizing *Mycobacterium tuberculosis* as a potential pathogen in respiratory infections, especially in the elderly during the COVID-19 pandemic, is critical. This case highlights the pivotal role of Gram staining in diagnosis, particularly when COVID-19 tests consistently show negative results. Recognition of Gram-ghost bacilli facilitated prompt tuberculosis diagnosis, emphasizing the enduring diagnostic value of Gram staining, especially in the COVID-19 era.

## Introduction

The term "Gram-ghost appearance" is used to describe the Gram-neutral or Gram-ghost (neither positive nor negative) appearance of mycobacteria when observed under Gram staining [1, 2]. It is important to consider *Mycobacterium tuberculosis* as a potential pathogen in acute respiratory infections, particularly in elderly patients, even during the COVID-19 pandemic. Due to the impact of the COVID-19 pandemic, access to detailed medical histories and physical examinations has become challenging [3]. Additionally, in cases where COVID-19-related tests yield negative results, observation over time often becomes a common approach. In this report, we present a case where a Gram stain played a crucial role in diagnosis, especially in a scenario where COVID-19 tests consistently showed negative results.

## Case presentation

During the COVID-19 pandemic, a 79-year-old Japanese man with hypertension visited an outpatient fever clinic with a one-week history of fever, cough, and sputum. His medications were amlodipine and doxazosin. Because he tested negative for SARS-CoV-2 on the polymerase chain reaction (PCR) test, he was treated with azithromycin for suspected acute bronchitis or pneumonia. However, his condition did not improve, and he was transferred to our fever clinic. He had no medical history of contact with tuberculosis, visits to prison, refugee camps, or substance abuse.

On physical examination, the patient appeared mildly ill; his blood pressure was 126/64 mm Hg, his pulse rate was 73 beats per minute and regular, his temperature was 37.6°C, his respiratory rate was 20 breaths per minute, and his peripheral arterial oxygen saturation was 98% on room air. Chest examination revealed coarse crackles in the right chest region. The remaining physical findings were unremarkable. Laboratory investigations showed a white blood cell count of 8,980/μL (normal range: 3,500-9,000/μL); hemoglobin of 12.3 g/dL (normal range: 13-17 g/dL); and platelet count: of 178,000/μL (normal range: 140,000-340,000/μL). Evaluation of serum chemistry revealed the following results: blood urea nitrogen: 19 mg/dL (normal range: 8.0-20.0 mg/dL); creatinine: 0.6 mg/dL (normal range: 0.6-1.0 mg/dL); sodium: 140 mEq/L (normal range: 0-0.2 mg/dL); potassium: 3.7 mEq/L (normal range: 0-0.2 mg/dL); chloride: 109 mEq/L (normal range: 0-0.2 mg/dL); albumin: 3.0 g/dL (normal range: 4.1-5.1 g/dL); total protein: 6.0 g/dL (normal range: 6.6-8.1 g/dL); aspartate aminotransferase: 30 IU/L (normal range: 13-30 IU/dL); alanine aminotransferase: 38 IU/L (normal range: 10-42 U/L); lactate dehydrogenase: 174 IU/L (normal range: 124-222 U/L); total bilirubin: 0.5 mg/dL (normal range: 0.4-1.5 mg/dL); glucose: 101 mg/dL (normal range: 73-109 mg/dL); and C-reactive protein: 4.6 mg/dL (normal range: 0-0.2 mg/dL) (Table 1).

**Table 1:** The patient's initial blood test results

Parameter	Result	Normal range
White blood cell count	8,980/μL	3,500-9,000/μL
Hemoglobin	12.3 g/dL	13-17 g/dL
Platelet	178,000/μL	140,000-340,000/μL
Blood urea nitrogen (BUN)	19 mg/dL	8.0-20.0 mg/dL
Creatinine	0.6 mg/dL	0.6-1.0 mg/dL
Sodium	140 mEq/L	135-145 mEq/L
Potassium	3.7 mEq/L	3.5-5.0 mEq/L
Chloride	109 mEq/L	98-107 mEq/L
Albumin	3.0 g/dL	4.1-5.1 g/dL
Total protein	6.0 g/dL	6.6-8.1 g/dL
Aspartate aminotransferase (AST)	30 IU/L	13-30 IU/L
Alanine aminotransferase (ALT)	38 IU/L	10-42 IU/L
Lactate dehydrogenase (LDH)	174 IU/L	124-222 IU/L
Total bilirubin	0.5 mg/dL	0.4-1.5 mg/dL
Glucose	101 mg/dL	73-109 mg/dL
C-reactive protein (CRP)	4.6 mg/dL	0-0.2 mg/dL

**Figure 1:**
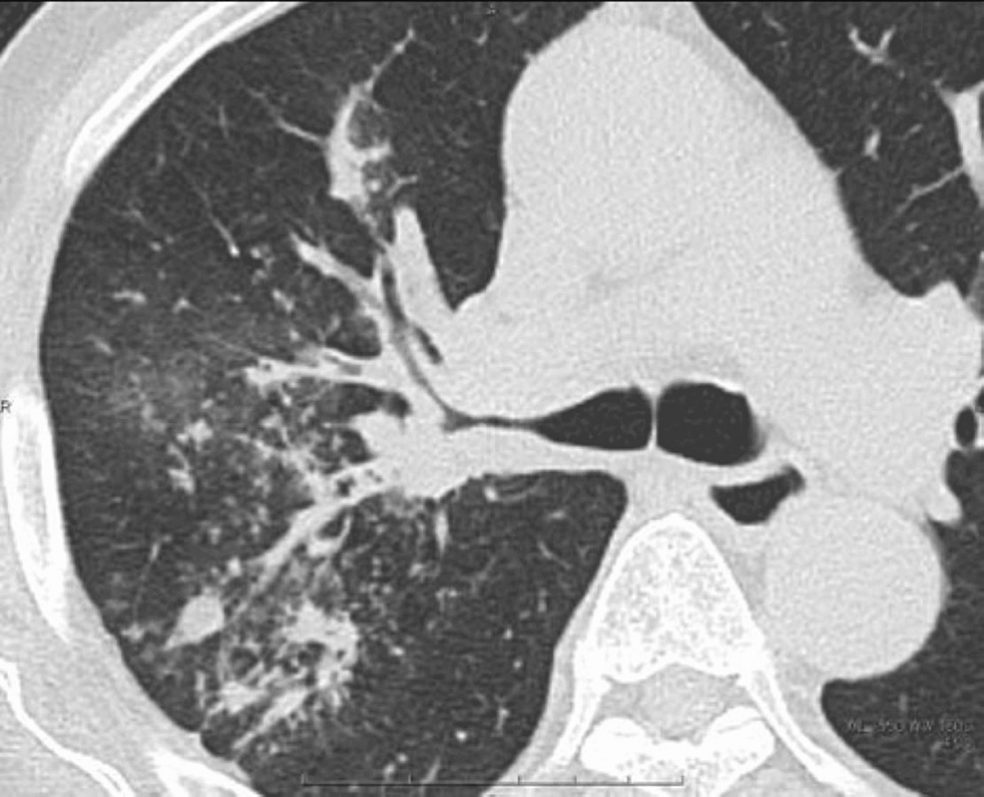
Computer tomography of the chest Computed tomography of the chest showed the bronchus of the upper lobe of the right lung to be thickening, with scattered mucous plugs and granular shadows or consolidation extending into the upper lobe.

Urinalysis was normal. In response to COVID-19 infection prevention measures, we conducted a direct computed tomography (CT) of the chest, which showed that the bronchus of the upper lobe of the right lung was thickening with scattered mucous plugs and granular shadows or consolidation extending into the upper lobe (Figure 1).

At our facility, GeneXpert testing was not conducted due to its unavailability. The initial, most likely diagnosis was acute bronchitis or mild community-acquired pneumonia at this point. After taking the culture of sputum, azithromycin was prescribed. The second SARS-CoV-2 PCR test also returned negative results.

**Figure 2:**
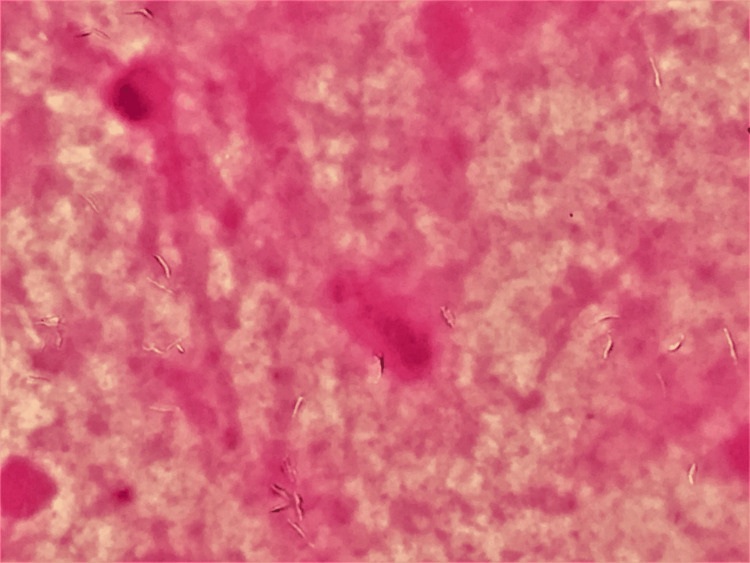
Gram staining of the sputum under appropriate focus (×1,000) Gram staining of the sputum revealed "Gram-ghost" bacilli, indicating a pulmonary acid-fast bacillus infection, where the observed transparent Gram-positive rods suggested mycobacterial involvement.

Gram staining is valuable for identifying mycobacteria and serves as an early diagnostic clue for mycobacterial infection. Gram staining of the sputum revealed Gram-ghost bacilli, indicating a pulmonary acid-fast bacillus infection (Figure 2).

**Figure 3:**
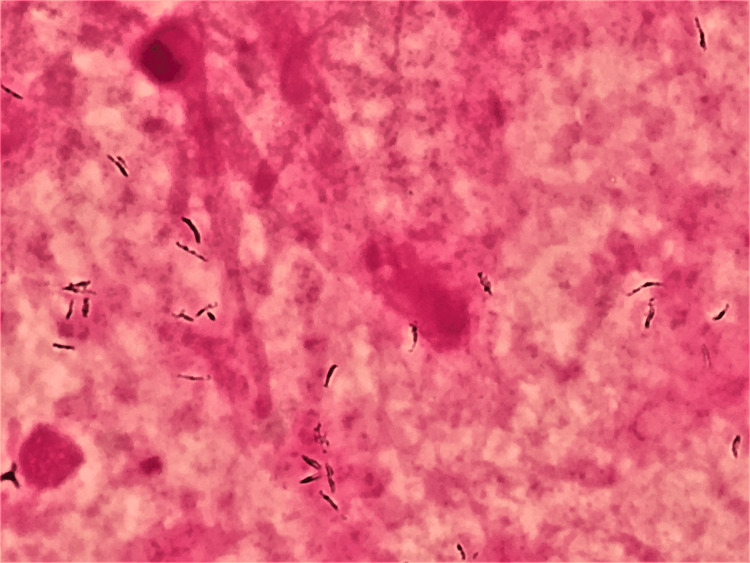
Gram staining of the sputum under a mildly longer focal distance (×1,000) Examination with a longer focal distance revealed weakly stained Gram-positive rods, providing additional insights into the bacterial morphology.

In the examination performed using a longer focal distance, weakly stained, Gram-positive rods were found (Figure 3).

**Figure 4:**
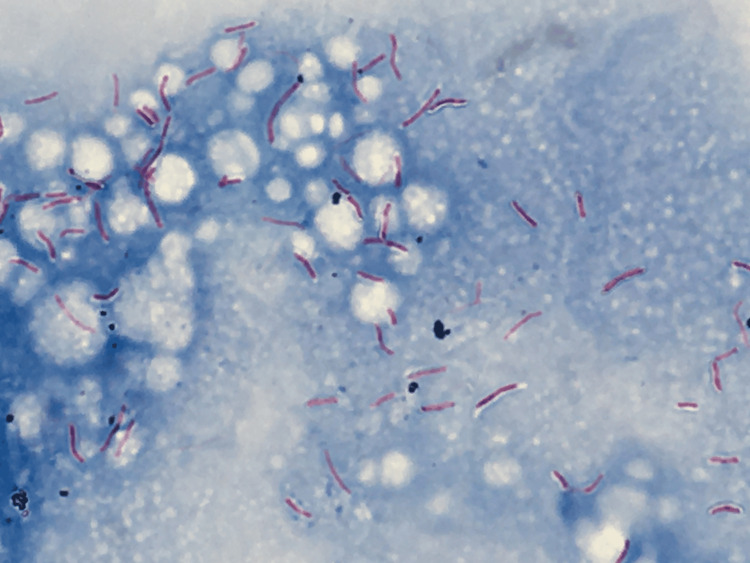
Ziehl-Neelsen staining under appropriate focus (×1,000) Ziehl-Neelsen staining confirmed the presence of acid-fast rods.

Ziehl-Neelsen staining confirmed the presence of acid-fast rods (Figure 4).

The *Mycobacterium tuberculosis* PCR test was positive. The culture results also confirmed the presence of *Mycobacterium tuberculosis*. The patient was transferred to a tuberculosis-specialized hospital and completed six months of treatment. Isoniazid, rifampin, pyrazinamide, and ethambutol were initiated for the first two months, followed by continued treatment with isoniazid and rifampin for an additional four months. All sensitivity results were susceptible.

## Discussion

In Japan, the crude incidence rate of tuberculosis decreased from 33.9 to 13.9 per 100,000 people. Similarly, the crude mortality rates decreased from 2.2 to 1.5 per 100,000 people, respectively [4]. Annual age- and sex-standardized notification rates of tuberculosis peaked at 599.0 per 100,000 population in 1955 and fell by 99% to 4.5 in 2022 [5]. In relation to tuberculosis and COVID-19, while numerous modelling studies assessing the COVID-19 pandemic's effect on tuberculosis have suggested the potential for thousands of additional tuberculosis-related deaths in the future, a recent review emphasized the lack of empirical evidence regarding the pandemic's impact on tuberculosis outcomes [6]. Indeed, an important question that remains unanswered is how the pandemic has affected the health outcomes of hundreds of thousands of people with undiagnosed tuberculosis in the years before and during the pandemic [7].

Gram staining is valuable for identifying mycobacteria and serves as an early diagnostic clue to mycobacterial infection. Gram-ghost appearance refers to the Gram-neutral or Gram-ghost (neither positive nor negative) appearance of mycobacteria on Gram staining [1, 2]. In several cases, Gram staining has been reported as a key diagnostic tool for mycobacterial infections [8, 9]. In Japan, in addition to Gram staining performed by clinical laboratory technicians, some internal medicine residents and infectious disease specialists can perform Gram staining [10]. Gram staining has two major advantages in the identification of mycobacteria [9]. First, it helps differentiate mycobacterial infections from community-acquired pneumonia by detecting Gram-ghost bacilli in initial specimens, thereby preventing inappropriate fluoroquinolone use and delays in the diagnosis of pulmonary tuberculosis. Second, it enables early identification of potential cases of pulmonary tuberculosis, facilitates timely isolation of patients to prevent airborne transmission, and ensures laboratory safety by implementing appropriate precautions during aerosol-generating procedures. Gram staining remains a powerful diagnostic tool, even during the COVID-19 pandemic.

## Conclusions

We reported a case where Gram staining proved to be valuable in the diagnosis of tuberculosis. Recognition of Gram-ghost bacilli on the initial Gram staining is still useful because it provides an early diagnostic clue of mycobacterial infection in situations where mycobacterial infection is not suspected. It is important to keep in mind that *Mycobacterium tuberculosis* can be a causative pathogen in community-acquired pneumonia, especially in elderly patients, even in the COVID-19 era.
